# Studying brain activity during word-by-word interactions using wireless EEG

**DOI:** 10.1371/journal.pone.0230280

**Published:** 2020-03-24

**Authors:** Tatiana Goregliad Fjaellingsdal, Diana Schwenke, Esther Ruigendijk, Stefan Scherbaum, Martin Georg Bleichner

**Affiliations:** 1 Department of Psychology, European Medical School, University of Oldenburg, Oldenburg, Germany; 2 Department of Psychology, Technische Universität Dresden, Dresden, Germany; 3 Cluster of Excellence Hearing4all, University of Oldenburg, Oldenburg, Germany; 4 Department of Dutch, University of Oldenburg, Oldenburg, Germany; University of Amsterdam, NETHERLANDS

## Abstract

We introduce here the word-by-word paradigm, a dynamic setting, in which two people take turns in producing a single sentence. This task requires a high degree of coordination between the partners and the simplicity of the task allows us to study with sufficient experimental control behavioral and neural processes that underlie this controlled interaction. For this study, 13 pairs of individuals engaged in a scripted word-by-word interaction, while we recorded the neural activity of both participants simultaneously using wireless EEG. To study expectation building, different semantic contexts were primed for each participant. Semantically unexpected continuations were introduced in 25% of all sentences. In line with the hypothesis, we observed amplitude differences for the P200—N400—P600 ERPs for unexpected compared to expected words. Moreover, we could successfully assess speech and reaction times. Our results show that it is possible to measure ERPs and RTs to semantically unexpected words in a dyadic interactive scenario.

## Introduction

Humans interact on a daily basis, using language to communicate. Such communications are marked by a constant back and forth between the speaker(s) and listener(s). The demands during such conversations are high. Each person in a conversation has specific conversational goals, i.e. a specific idea she wants to convey. To converse, one has to plan the general line of argumentation, construct individual sentences and utter individual words. At the same time, one has to adjust dynamically dependent on the other person’s behavior and replies. Smooth alternations between speaker and listener indicate an effective dynamic. A dynamic that is facilitated by predicting what other people will say and using it to prepare our response accordingly [1]. This often works out and allows us to quickly respond, and even initiate an adequate response before the other person has finished the sentence [2]. However, the other person might say something, which we did not expect. In these situations, we go through a cascade of processes. First, we realize that what the other person said does not match with our expectation, then that we have to refrain from uttering our planned response, and finally we have to generate a more fitting response. In our research, we are interested in understanding these dynamic processes and their underlying neural mechanisms. Here, we present a paradigm that allows to assess these processes with wireless EEG.

The study of neural correlates in individuals has provided an invaluable basis for our understanding of linguistic processes [3]. The challenge now lies in relating these processes to their application in real life interactive scenarios [4]. Latest methodological developments in neuroscientific research allow us to study the interaction of two persons in more dynamic scenarios [5]. The complexity of interactions, with the constant back and forth between interacting partners and the need to plan and to adapt adequate responses to the other person’s utterances, demands new experimental paradigms that combine this openness with the necessary experimental control. Here, we present a step towards studying neural processes that underlie interactive language use, narrowing the gap between EEG studies in isolated individuals and interacting individuals.

Due to its high temporal resolution, electroencephalography (EEG) has been widely used to assess the neural underpinnings of language. Linguistic neural correlates have been primarily assessed in individuals seeing one word at a time on a screen or listening to pre-recorded speech streams [6–10]. Established linguistic event-related potentials (ERPs), the averaged EEG response time-locked to the onset of words of interest, include for instance the N400 and P600—which show amplitude modulations between words that are congruent or incongruent within a sentence context, e.g., ‘*The coffee is too hot to* ***drink*’** vs. ‘*The coffee is too hot to* ***cry*’** [10–14]. Importantly, these modulations are also apparent if the word is not wrong, but merely unexpected within the context [15].

To study these linguistic neural correlates not only in isolated individuals but also during interaction with others is challenging. Social interactions are very diverse and open-ended. The study of neurophysiological processes, on the other hand, requires a high degree of experimental control to ensure analyzable and interpretable data. The open nature of social interactions and the necessary experimental control are seemingly mutually exclusive. In open interactions, we do have multiple methodological challenges: (i) the variability of the language stimulus material, (ii) the muscular artifacts in the EEG signal induced by speaking, and (iii) the mapping of the critical utterances to the segment in the brain data. The result is therefore often a tradeoff between naturalness and control.

To address the mentioned aspects, we introduce a paradigm that we have borrowed from improvisational theater: the word-by-word game. This game comprises all processes involved in the back and forth of a conversation in a very condensed and controlled form that lends itself for studying the underlying neural processes. The game itself provides an interesting model of how interactions can take place: a joint action that requires two participants to coordinate their utterances to form meaningful sentences together taking turns for each word. It resembles natural interactions in its coordinative nature, the building of expectations, the hesitations after unexpected events, language production, language comprehension, and turn-taking.

In the word-by-word game, two people jointly generate sentences taking turns in saying the next word, without having additional means to coordinate their actions. When people play the game it is interesting to see that some people can jointly produce syntactically and semantically correct sentences. The words are fluently strung together by the two. However, one can also observe long hesitations when one person encounters an unexpected word. The words can be so unexpected that the person cannot come up with any word at all. Interestingly, the successful joint sentence construction can be practiced. While novice players may not be able to construct a single coherent sentence, experienced players can be so fluent, that they can even interact with a third person in a conversation.

A first study using this approach comes from Himberg and colleagues [16], who targeted temporal synchronization of persons during such an interactive joint word-by-word story telling. In this study, the participants were completely free in their interaction, without any constraints (apart from a time limit). While this setup allows for behavioral analysis, the completely open nature makes it suboptimal as an EEG paradigm. To study the neural underpinnings of verbal interactions, increased experimental control is necessary. EEG, as any other imaging modality, provides certain constraints on possible paradigms that need to be considered: first, movements need to be limited to avoid artifacts, and second, the necessary signal averaging requires a number of repetitions of events ideally in an equal proportion for different conditions. For this, it needs to be assured that the events of interest occur sufficiently often. Consequently, a more controlled version of the word-by-word paradigm would allow studying its interactive elements whilst keeping a sufficiently high degree of experimental control.

To assess verbal interactions, we here propose a more constrained version of the word-by-word game [16] that allows for maximal control. The game itself provides an interesting model of how interactions can take place: a joint action that requires two participants to coordinate their utterances to form meaningful sentences together taking turns for each word. It resembles natural interactions in its coordinative nature, the building of expectations, the hesitations after unexpected events, language production, language comprehension, and turn-taking. It is by definition an interactive task of linguistic nature, where two persons have to adapt to each other to form a coherent sentence. From an experimental point of view, this paradigm provides many possibilities to manipulate in how far the participants share a joint goal. The joint sentence production is doomed to fail, if the partners do not share a common mindset about the utterance they want to produce. That is, if one of the partners beliefs that the joint objective is to buy a book, while the other assumes the objective is to buy a drink. The clear structure (word-by-word) further allows defining precise onsets of events for EEG ERP analysis. This ultimately allows relating the new finding to a large body of literature. To be able to link established knowledge of language processing to findings within such a new paradigm, we developed a systematic approach to target established linguistic components within a word-by-word interaction. The focus lies on well-established ERPs linked to expectation building—the P200 and N400 ERP [17]. The positivity around 200 ms after word onset has been described in studies contrasting expected and unexpected words within a given context [17,18]. Similarly, the N400 ERP has a strong link to the predictability of a word, where its amplitude modulation reflects the expectancy violation [7,10,12,13,17,19]. Therefore, we study the feasibility of recording these two ERP components linked to expectation building while actively engaged in interaction.

In the present setup, neurophysiological responses to expectation violations during dyadic word-by-word interactions were measured with wireless EEG. Wireless EEG allows to study neural processes beyond the lab [20–22]. Eventually, the word-by-word game could be studied using such a wireless EEG system (as opposed to a classical wired EEG system) during a live interaction on stage. Here, we present the first step towards that goal, to learn how far the active involvement of the participants (e.g., talking) opposes the analysis of ERP components [22–24].

For both partners of an interacting pair, we manipulated the expectation for specific words in a sentence. For the neural responses, we hypothesized an increased N400 ERP amplitude to unexpected compared to expected words ([8–10,25–27]; but see new discussions on prediction in language, e.g., [28]). In addition, we hypothesized a similar P200 ERP effect, an increased P200 amplitude for unexpected words, according to previous findings [17,18]. Behaviorally, we hypothesized an increase in response times (i.e., reading aloud times) after an unexpected word in line with observations of the game and self-paced reading studies [6]. Assessing whether well-understood neural processes are detectable in dynamic and interactive, yet controlled setups is an important step towards the study of neural processes in open social interactions.

## Methods

### Participants

Twenty-six healthy German-native speakers (mean age 24.4 years, 15 females, 25 right-handed) took part in this study. Two interacting participants were measured simultaneously as a pair per session. All had normal or corrected-to-normal vision. Exclusion criteria for participation were language disorders and neurological or psychiatric diseases. Participants were recruited via the online notice student board and received monetary compensation for participation (€ 8,- per hour). Monetary compensation was not dependent on their performance. Participants were not matched or balanced for gender. The study was approved by the local ethics committee of the University of Oldenburg. All participants signed written informed consent prior testing according to the declaration of Helsinki.

### Paradigm

**Fig 1:**
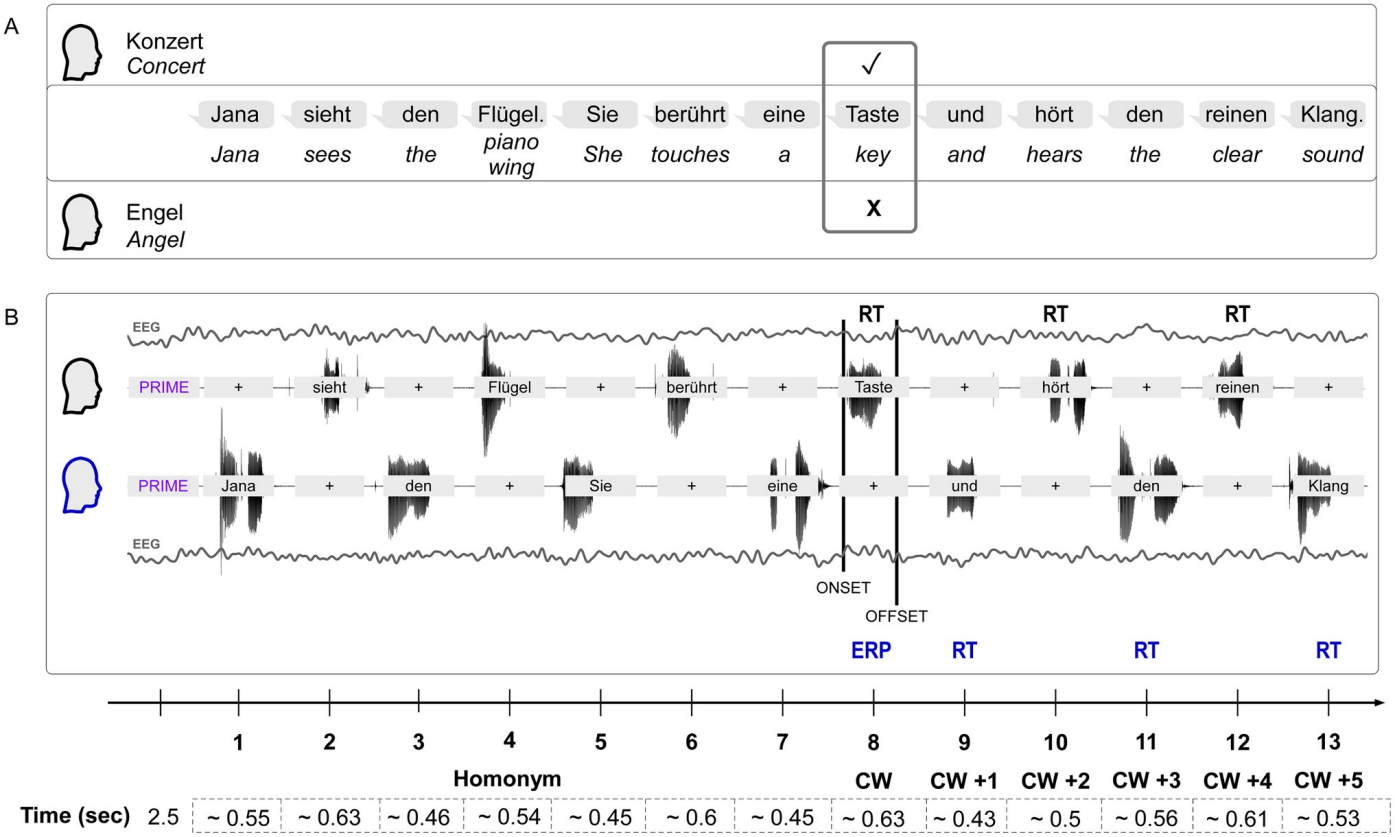
Experimental paradigm and setup. (A) Paradigm: Two example reference frames for each participant of a pair are shown. The prime for each participant would define a meaning context for the homonym (*Flügel—piano/wing*) in the upcoming sentence. In this example, one had a prime (*Konzert—concert*) for one meaning (piano) and the other participant had a prime (*Engel—angel*) for the other meaning (wing) of the homonym. The sentence meaning was ambiguous and fit to both reference frames until the critical word (*Taste—key*), which would fit the reference frame of a piano (i.e., it would be congruent to concert), but not the reference frame of a wing (i.e., it would be incongruent to angel). Sentence translation: ‘*Jana sees the piano/wing*. *She touches a key and hears the clear sound*.’ (B) Setup: EEG and audio were recorded from both participants concurrently. The prime had to be read silently and was shown in a violet color to distinguish it more easily from the words of the sentence shown in black that had to be read aloud. Each participant started to read aloud Word 1 of the sentence in 50% of the trials. The analysis focused on the ERP of the CW (highlighted in blue) that was measured for the participant (blue head) that perceived the word read aloud by the interacting partner (black head). The offline defined speech onset of the interacting partner was used as onset marker for the listened word. The reaction time (RT) to speak was measured for CW+1, CW+3, and CW+5 in the same participant after the ERP of the CW. In the same trial, the RT to speak the CW, CW+2, and CW+4 was measured in the partner. In the experiment, the prime was either congruent for both participants or congruent for one participant and incongruent for the interacting partner. Presentation time of the prime and mean speech times of each word are shown. (RTs are not included).

Here we study expectation violations during word-by-word interactions in a pair of participants. We constructed the stimulus material such that the data of both participants could be analyzed. The two participants jointly had to read out sentences. Prior to reading the sentences, they were primed to a specific context, which either matched or did not match the following sentences. The sentences included homonyms (one word with at least two different meanings) and were constructed such that they were congruent to both contexts up to the eighth word. The eighth word (from now on critical word or CW) in the sentence was then expected or unexpected within the personal context (compare example 1 and Fig 1A).

(1) Prime 1 (congruent to CW) Konzert (concert)
  Prime 2 (incongruent to CW) Engel (angel)
  *Jana sieht den Flügel*. *Sie berührt eine* ***Taste***, *und hört den reinen Klang*.
  *(Jana sees the piano/wing*. *She touches a* ***key*** *and hears the clear sound*.)

To allow for sufficient experimental control, the sentences were predefined. The material was selected such that out of 240 trials, 75% would be congruent and 25% would be incongruent for each participant. The prime was either always congruent for both participants or congruent for one of the participants and incongruent for the other. Each participant was the producer of the critical word in 120 trials and the perceiver of the critical word in the other 120 trials. The main contrast of neural responses was calculated for the critical words that were perceived (compare Fig 1B). Further details of trial construction can be found in the supporting information—Paradigm—Trial construction.

A trial started with the presentation of a fixation cross for 0.5 seconds and the violet-colored prime word for 2.5 seconds on gray screens for both participants. The first black-colored word of the sentence was presented in the center of the screen of the participant who started reading aloud; at the same time, the second participant saw a black fixation cross in the center of the screen (compare Fig 1B). When she finished reading aloud this first word, the second word of the sentence was presented on the screen of the other participant. As soon as he would finish reading this word, the third word was presented on the screen of the first participant and so on. This word-by-word turn taking continued until the thirteenth, final word of the sentence. A blank gray screen was presented for 1.5 sec between trials. The participants could not see the other persons screen. Prime and trial words were shown in different colors to simplify their distinction preventing that the prime was read out aloud by mistake. The experiment was programmed with psychophysics toolbox [29,30] in Matlab R2012b.

### Language material

For the sentences, 240 German homonyms (partly from [31]) were used. One noun prime was selected for each meaning of the homonym (i.e., two primes in total per sentence). Homonyms allow maintaining a semantic context (i.e., a sentence) ambivalent up to a critical word (CW) clarifying the meaning of the homonym. Employing different primes for one participant vs. the other participant further allows to provide different contexts for the interacting pair, rendering the same critical word expected for one participant and unexpected for the other participant given a specific context.

One trial consisted of 13 words in two sentences, where the first sentence was a four-word main clause (*name—verb—determiner—homonym*) and the second sentence a four-word main clause (*pronoun—verb—determiner—noun*) followed by a five-word subordinate clause or a one-word conjunction with a four-word main clause. The homonym was always the 4^th^ word and both meanings of the homonym were plausible until the 8^th^ word, i.e., the critical word (CW). We constructed the sentences so that a CW (2a: *Taste*—*key*; 2b: *Feder*—*feather*) for either meaning of the homonym (2a: *Flügel*—*piano*; 2b: *Flügel*–*wing*) would fit into the sentence (see examples 2 & 3), disregarding the words after the CW. In the experiment, only the first sentence option (2a & 3a) was used (since congruency was purely defined by the prime before the sentence).

(2a) *Jana*_1_ *sieht*_2_ *den*_3_ ***Flügel***_**4**_. *Sie*_5_ *berührt*_6_ *eine*_7_ ***Taste***_**8**_, *und*_9_ *hört*_10_ *den*_11_ *reinen*_12_ *Klang*_13_.
  *(Jana*_1_ *sees*_2_ *the*_3_ ***piano***_**4**_. *She*_5_ *touches*_6_ *a*_7_ ***key***_**8**_ *and*_9_ *hears*_10_ *the*_11_ *clear*_12_ *sound*_13_.)
(2b) *Jana*_1_ *sieht*_2_ *den*_3_ ***Flügel***_**4**_. *Sie*_5_ *berührt*_6_ *eine*_7_ ***Feder***_**8**_ *…*.
  *(Jana*_1_ *sees*_2_ *the*_3_ ***wing***_**4**_. *She*_5_ *touches*_6_ *a*_7_ ***feather***_**8**_ *…*.)
(3a) *Peter*_1_ *sucht*_2_ *eine*_3_ ***Bank***_**4**_. *Er*_5_ *braucht*_6_ *etwas*_7_ ***Ruhe***_**8**_, *um*_9_ *später*_10_ *ausgehen*_11_ *zu*_12_ *können*_13_.
  *(Peter*_1_ *searches*_2_ *a*_3_ ***bench***_**4**_. *He*_5_ *needs*_6_ *some*_7_ ***rest***_**8**_ *to be able to go out later*.)
(3b) *Peter*_1_ *sucht*_2_ *eine*_3_ ***Bank***_**4**_. *Er*_5_ *braucht*_6_ *etwas*_7_ ***Geld***_**8**,_ *…*.
  *(Peter*_1_ *searches*_2_ *a*_3_ ***bank***_**4**_. *He*_5_ *needs*_6_ *some*_7_ ***money***_**8**_ *…*.)

Congruency was solely defined by the prime presented prior to the sentence. So the sentence in examples 4 and 5 was either presented with a congruent prime (4: *Konzert*; 5: *Park*) or an incongruent prime (4: *Engel*; 5 *Kredit*) to the CW (4: *Taste*; 5: *Ruhe*). Further details of sentence construction can be found in the supporting information—Paradigm.

(4) Congruent Prime Konzert (concert)
  Incongruent Prime Engel (angel)
  *Jana*_1_ *sieht*_2_ *den*_3_ ***Flügel***_**4**_. *Sie*_5_ *berührt*_6_ *eine*_7_ ***Taste***_**8**_, *und*_9_ *hört*_10_ *den*_11_ *reinen*_12_ *Klang*_13_.
  *(Jana*_1_ *sees*_2_ *the*_3_ ***piano***_**4**_***/wing***_**4**_. *She*_5_ *touches*_6_ *a*_7_ ***key***_**8**_ *and*_9_ *hears*_10_ *the*_11_ *clear*_12_ *sound*_13_.)
(5) Congruent Prime Park (park)
  Incongruent Prime Kredit (credit)
  *Peter*_1_ *sucht*_2_ *eine*_3_ ***Bank***_**4**_. *Er*_5_ *braucht*_6_ *etwas*_7_ ***Ruhe***_**8**_, *um*_9_ *später*_10_ *ausgehen*_11_ *zu*_12_ *können*_13_.
  *(Peter*_1_ *searches*_2_ *a*_3_ ***bench/bank***_**4**_. *He*_5_ *needs*_6_ *some*_7_ ***rest***_**8**_ *to be able to go out later*.)

### EEG recording

Two wireless EEG systems were used to record brain electrical activity. The wireless setup allows more mobility than non-wireless EEG setups, e.g., for participants to move and stand up during breaks. The system consists of a small wireless amplifier (Smarting, www.mBrainTrain.com, Belgrade, Serbia) which is attached to the back of the EEG cap (Easycap, Herrsching, Germany). The cap has 26 sintered Ag/AgCl electrodes (international 10/20: Fp1, Fp2, F7, Fz, F8, FC1, FC2, C3, Cz, C4, T7, T8, TP9, TP10, CP5, CP1, CPz, CP2, CP6, P3, Pz, P4, O1, and O2, reference: FCz, ground: AFz). The EEG data were sent wirelessly to a recording computer via Bluetooth, received by the amplifiers acquisition software (Smarting Software 2.0.0, Smarting mBrainTrain, Belgrade, Serbia) and then sent as an LSL stream. The EEG data was sampled with a sampling rate of 500 Hz. Electrode impedances before measurement were below 10 kΩ.

The utterances of both participants were recorded with a stereo microphone with a sampling rate of 44100 Hz. The two EEG streams and respective two audio streams, as well as the marker stream, were recorded synchronously with the Lab Recorder of Lab Streaming Layer [32].

### Procedure

Prior invitation to testing, a telephone interview was conducted with each participant to ensure that all inclusion criteria were met (see Section 2.1). Two participants (one pair) were invited per session. We controlled that the two participants did not know each other by asking them.

After signing informed written consent, each participant completed the Edinburgh Handedness Inventory [33] and a common state questionnaire. After the EEG caps were fitted (see Section 2.4), participants were seated in the same laboratory in front of two computer screens. Two microphones (ETM-006, Lavalier Microphone) with audio pop shields attached to a tripod were placed near their mouths (see S1 File of S1 Fig).

An online voice-key in the experiment detected speech onsets and offsets of each participant. The voice-key triggered the presentation of the next word to the other participant. The voice-key was calibrated before the experiment to find the optimal volume threshold for each individual.

For the experiment, participants were instructed to read silently the prime and to read aloud each word that was visible on their screen (see Fig 1B). They were told to avoid task unrelated movements and to use inter-trial intervals (blank screen between trials) for necessary movements. A short test run with six training sentences was completed to clarify the structure and task to the participants and then the experiment started. Every 15 trials there was a pause.

To ensure that the participants’ were paying attention to the primes and sentences, a multiple choice control task was applied in each pause. The task comprised two questions, where the participants had to mark (1) a prime that was presented within the previous block and (2) the answer to a semantic question about one of the sentences presented in the previous block. Only congruent primes and sentences were included for questioning, to avoid a focus on incongruent trials. Correct responses here pointed to an effective semantic analysis of primes and following sentences.

After the experiment, participants were asked to fill out an evaluation questionnaire. Results can be found in the supporting information—Evaluation results.

### Audio preprocessing

The utterances of one participant were used as (heard) stimuli for the other participant (compare Fig 1B). For this purpose, the speech onset times that were marked during the experiment (voice-key 100 ms resolution) were determined more precisely offline for the ERP analysis.

For this purpose, the audio signal was epoched around the online onset marker (-0.5 sec to 1 sec) to access each single word. To erase low frequency fluctuations the audio signal was highpass filtered (FIR filter) at 35 Hz. Thereafter, the signal was downsampled to 1470 Hz. As a next step, the envelope of the audio signal was computed (filter length 300) and lowpass filtered at 730 Hz (Butterworth FIR filter, -6 dB). Further, the cepstrum [34] of the downsampled audio signal was calculated using the ‘melcepst’ function [35]. The first and second fundamental frequencies of the cepstrum were extracted and lowpass filtered at 600 Hz. The root mean square (RMS) of the downsampled audio signal was then computed and the function ‘findchangepts’ (MATLAB toolbox signal processing) applied. The output of this function are a series of markers showing abrupt changes in the RMS signal. If these markers indicate the real onset of speech in the audio signal, was evaluated by using the previously computed envelope and the first and second fundamental frequencies of the cepstrum. Only if changes were apparent in the envelope and cepstrum too, the onset marker was accepted. Hereby, false positive onsets through lip smacks or other artifacts were prevented. The newly calculated speech onset points were used as event markers in the EEG data sets (see Fig 1B). For the speech offset detection the same procedure was applied but with a time-reversed audio signal.

### Behavioral analysis

Response accuracy for the multiple choice control task was calculated for each participant for the 32 questions (two after each block).

For descriptive purposes, mean speech times for each spoken word (1–13) and each participant were extracted from the corrected audio data. Average sentence production time was calculated for each trial. Additionally, the word length, defined as number of letters, was calculated for each presented word.

The reaction time (RT) to speak, defined as the time that elapsed between the presentation of a word on the screen and a person’s speech onset of this word, was calculated for each word in a sentence (i.e., spoken words 1–13, where CW to CW+5 was split into congruent/ incongruent conditions; compare S1 Table). RTs smaller than 300 ms and greater than 1000 ms (2.6% of all words) were excluded from further analysis. Trials where the offline analysis of the speech offset showed that the preceding spoken word had not been terminated prior to the visual presentation of the word were excluded (4.14% of all words, 4.2% of words 8 to 13).

Participants took turns reading out each word of a sentence. Congruency was defined by the prime at the beginning of each trial (which could be congruent for both or incongruent for one and congruent for the other participant). Statistical analysis of the RT to speak for each congruency condition was therefore split into the two consecutive spoken word sequences: (1) CW, CW+2, and CW+4 and (2) CW+1, CW+3, and CW+5 (compare examples in Table 1 below and see Fig 1B and S1 Table for details of trial composition).

**Table 1:** 

	*Jana sieht den Flügel*. *Sie berührt eine Jana sees the piano/wing*. *She touches a*	*Taste*, *key*	*und and*	*hört hears*	*den the*	*reinen clear*	*Klang*. sound.
P1	Congruent Prime: *Konzert (concert)*	CW		CW+2		CW+4	
P2	Congruent Prime: *Konzert (concert)*		CW+1		CW+3		CW+5
	*Peter sucht eine Bank*. *Er braucht etwas*	*Ruhe*,	*um*	*später*	*ausgehen*	*zu*	*können*.
	*Peter searches a bench/bank*. *He needs some*	*rest*	*to be*	*able*	*to go*	*out*	*later*.
P1	Incongruent Prime: *Kredit (credit)*		CW+1		CW+3		CW+5
P2	Congruent Prime: *Park (park)*	CW		CW+2		CW+4	

To account for non-normally distributed RT data and the given word order, the effect of congruency of the sentence on the RTs to speak was analyzed with a generalized linear mixed model (GLMM; [36–38]) using the lme4 package [39] in R [40]. Linear Mixed Models (LMMs) are robust enough to contrast repeated measurement conditions with differing trial ratios [41–43]. Moreover, Generalized Linear Mixed Models (GLMMs) allow to account for non-normality of the response variable by adding a probability distribution. Here, we therefore used a GLMM with a gamma probability distribution and its default inverse link function [43]. The model was fit with the two fixed factors congruency and spoken word position in sentence and the two random intercepts participant and word length. Word length was added as a random factor since we expect it to be related to the (random) item variability. Item as such was not added as a random factor due to the correlation with the congruency condition. Two separate models were calculated: one for the sequence CW+1, CW+3, CW+5 and one for the sequence CW, CW+2, CW+4. Model fit was assessed with likelihood ratio tests via ANOVA comparing the original model with simpler models (i.e., without fixed factor A or B).

### EEG analysis

The usage of two wireless Bluetooth-based amplifiers recording at 500 Hz led to instances of data loss during recording. Details of the employed EEG data marker correction can be found in the supporting information—Package loss correction.

EEG data were preprocessed using EEGLAB [44] in Matlab R2016a. Artifact Subspace Reconstruction (ASR; [21,45]), as implemented in the clean_data EEGLAB plugin with default settings, was used for artifact preprocessing to deal with muscular artifacts, originating for example from eye blinks and jaw movements. ASR uses clean reference data (either acquired before the measurement or identified in the continuous data) to define windows, where deviations from the mean are apparent. These deviant windows are treated as missing data and reconstructed in the principal component subspace [45]. The ASR approach has been tested to successfully remove transient and large-amplitude artifacts in EEG signals [46], as present in this study. Previously to applying ASR, we filtered the data between 0.1 and 30 Hz (Finite impulse response filter (FIR), window type “Hann”, cutoff frequency −6 dB) and run the ‘clean artifacts’ function with default settings incorporated in the ASR package. This function scans the data for bad signal channels, drifts and bursts. Bad channels were excluded prior to ASR application.

For ERP analysis, the preprocessed and corrected EEG data were re-referenced to average mastoids (TP9 and TP10). Missing channels were interpolated using the spherical interpolation method of EEGLAB. Data were then epoched from -500 ms to 1500 ms to (i) heard words—the speech onset of the partner (i.e., heard word 1–7, heard word 8–13 congruent/incongruent), (ii) spoken words—the speech onset of the participant, and (iii) visual words—the visual presentation of the word on the screen of the participant. The focus of analysis lied on the heard words, while spoken CWs and visual CWs are visualized in the supporting information—ERP data, S3 File of S3 Fig. An automatic epoch rejection was applied, where all epochs exceeding three standard deviations from the mean signal were excluded from further analysis. Epochs were baseline corrected from -100 ms to 0 ms. To address concerns of baseline influences on the reported effects (compare [47,48], but see also [49,50]), we compared effects of the CW with and without baseline correction. There was no qualitative difference in the results, neither for the direction of the effects nor their significance.

To account for high inter-trial variability in the present paradigm, we applied Residue Iteration Decomposition (RIDE; [51]) on the EEG data of the heard words. The RIDE algorithm decomposes the ERP into the underlying components, relying on a stimulus-locked, a central cognitive, and a(n optional) response cluster (i.e., the classical assumption of a single trial). Here, we defined a stimulus-locked and two cognitive components without a response cluster, since the current setup does not require an overt response from the participant to the stimulus (e.g., a mouse click). The RIDE output is a latency-locked rather than a stimulus-locked ERP. This is achieved for each defined cluster by finding single trial peaks and re-synchronizing the peaks iteratively per trial. “Specifically, all single trials [are] synchronized to the detected single trial latencies by temporally shifting each trial with the relative lag between its latency and the median of all latencies across trials [52].” RIDE was run with a stimulus-locked component from 0 to 250 ms (the N100), a cognitive component from 150 to 350 ms (the P200), and a second cognitive component from 300 to 700 ms (the N400). The final output is a reconstructed ERP (without single trial information), where the defined components have been latency corrected. For the CW, the same number of trials were compared between congruent and incongruent conditions. The mean number of trials of the CW entering statistical analysis was 37 ± 1.9 for the congruent condition and 37 ± 2.6 for the incongruent condition.

After visual inspection of the RIDE-reconstructed CW ERP, we conducted a 2 x 4 repeated-measures ANOVA in SPSS (IBM Corporation, New York, USA) separately for each component: the P200, N400, and P600. Time windows of mean amplitudes were defined by visual inspection of the grand average over conditions, as well as based on prior studies (e.g., [17]). For the P200 we calculated the mean amplitude from 166 to 336 ms, for the N400 from 350 to 500 ms, and for the P600 from 500 to 650 ms. The main two-level factor of analysis was congruency (congruent, incongruent). To assess topographical distributions of the respective components, two separate repeated-measures ANOVAs were conducted (compare [25]: (1) an ANOVA for midline electrodes with the additional four-level factor electrode (Fz, Cz, CPz, Pz) and (2) an ANOVA for lateral electrodes with the additional four-level factor quadrant with an average of electrodes (left anterior: Fp1, FC1, F7, C3; left posterior: CP1, CP5, P3, O1; right anterior: Fp2, F8, FC2, C4; right posterior: CP2, CP6, P4, O2).

In a descriptive approach, we tested the presence of an N100 visible in the ERP of the 1^st^ word, indexing auditory analysis, against succeeding words 2, 3, and 4 (four level factor word) in an additional repeated measures ANOVA along the midline electrodes (four level factor electrode: Fz, Cz, CPz, Pz).

Greenhouse-Geißer corrected p-values with original degrees of freedom are reported, whenever Mauchly’s test indicated a violation of sphericity. Effect sizes are reported as partial eta squared values.

## Results

### Behavioral results

Participants’ responses to the multiple choice control task were correct in 81% (26 ± 3 out of 32 questions) on average, confirming that the participants did the task. For the questions regarding the prime, the average accuracy was 81% (13 ± 2 out of 16 questions). For the questions about the sentences, the average accuracy was 88% (14 ± 2 out of 16 questions).

**Fig 2:**
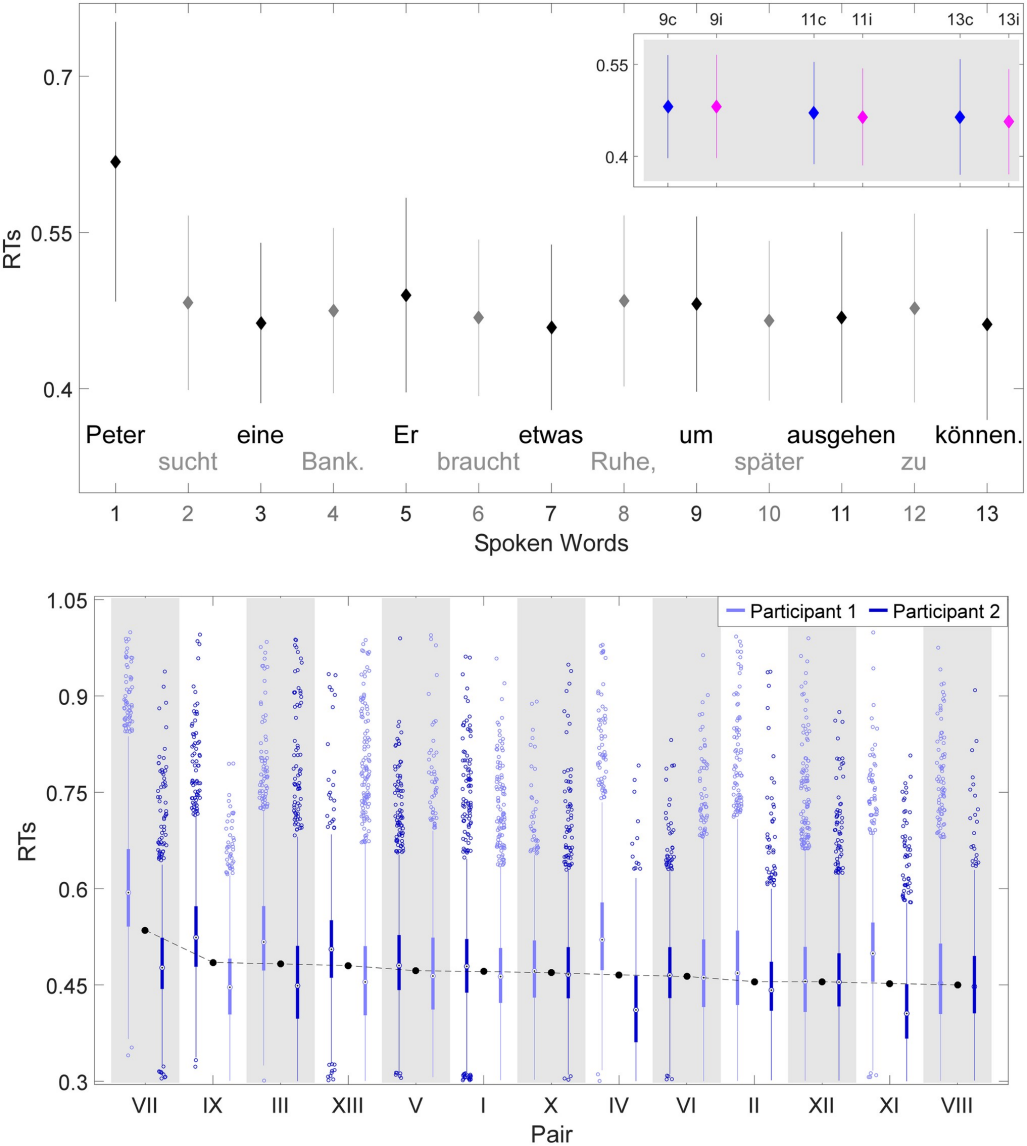
Behavioral results: Speech times and reaction times. (A) Grand average speech times for the spoken words 1 to 13. Error bars show standard deviations. Average word length (number of letters) is shown for each word. The sentences were uttered by both participants taking turns for each word: e.g., the black colored words by one participant and the grey colored words by the other participant. (B) Grand average reaction times (RTs) to speech onset—from visual presentation to speech onset—for the spoken words 1 to 13. Vertical bars represent standard deviation. One trial was produced by both participants: e.g., the black colored odd words by one participant, whereas the grey colored even words by the other participant. A generalized linear mixed effects model (GLMM) was computed for CW+1, CW+3, CW+5 (on gray background), that are split into congruent (blue dots) and incongruent conditions (magenta dots) for the participant speaking. Sentence translation: ‘Peter searches a bench. He needs some rest, to be able to go out later.

To complete the trial sentences a pair took on average 16 ± 2 seconds (excluding outliers ± 2.5 *SD* from the mean). Thereof approximately 7 seconds were articulations of words. The grand average speech time of a single word was 0.54 ± 0.07 seconds, but varied dependent on the word sentence position and respective word length, i.e., its letters (compare Fig 2A). On average, the time from visual presentation of the word to speech onset was 0.49 ± 0.1 seconds. It is apparent from the RTs to speech onset for each word of a sentence (see Fig 2B) that participants were comparatively slower to utter the first word of each sentence.

The effect of congruency on the RT to speech onset was analyzed with a generalized linear mixed model (GLMM) including the fixed factors congruency and word position. For sequence CW+1, CW+3, CW+5, congruency (congruent vs. incongruent) significantly affected RTs to speech onset [χ2 (1) = 7.58, *p* = .006]. Results of the model showed a mean predicted RT of 0.709 seconds and the predicted effect of congruency was to decrease the mean RT to 0.698 seconds (from congruent to incongruent condition). Word position in sentence (CW+1 vs. CW+3 vs. CW+5) was also of significant impact for the RTs to speech onset [χ2 (1) = 260.71, *p* < .0.001]. For sequence CW, CW+2, CW+4, congruency (congruent vs. incongruent) did not significantly affect the RTs to speech onset [χ2 (1) = 2.23, *p* = .136]. However, word position in the sentence (CW vs. CW+2 vs. CW+4) had a significant effect on RTs [χ2 (1) = 12.30, *p* < .001]. A full overview of the models output can be found in supporting information—GLMM results.

### EEG results

The word-by-word paradigm allows analyzing each sentence as a construct of both participants. Main analysis was performed on the CW ERP extracted as a response to the heard word, i.e., to the word uttered by the other participant. (A broader overview is given in the supporting information—ERP data, S3 File of S3 Fig).

**Fig 3:**
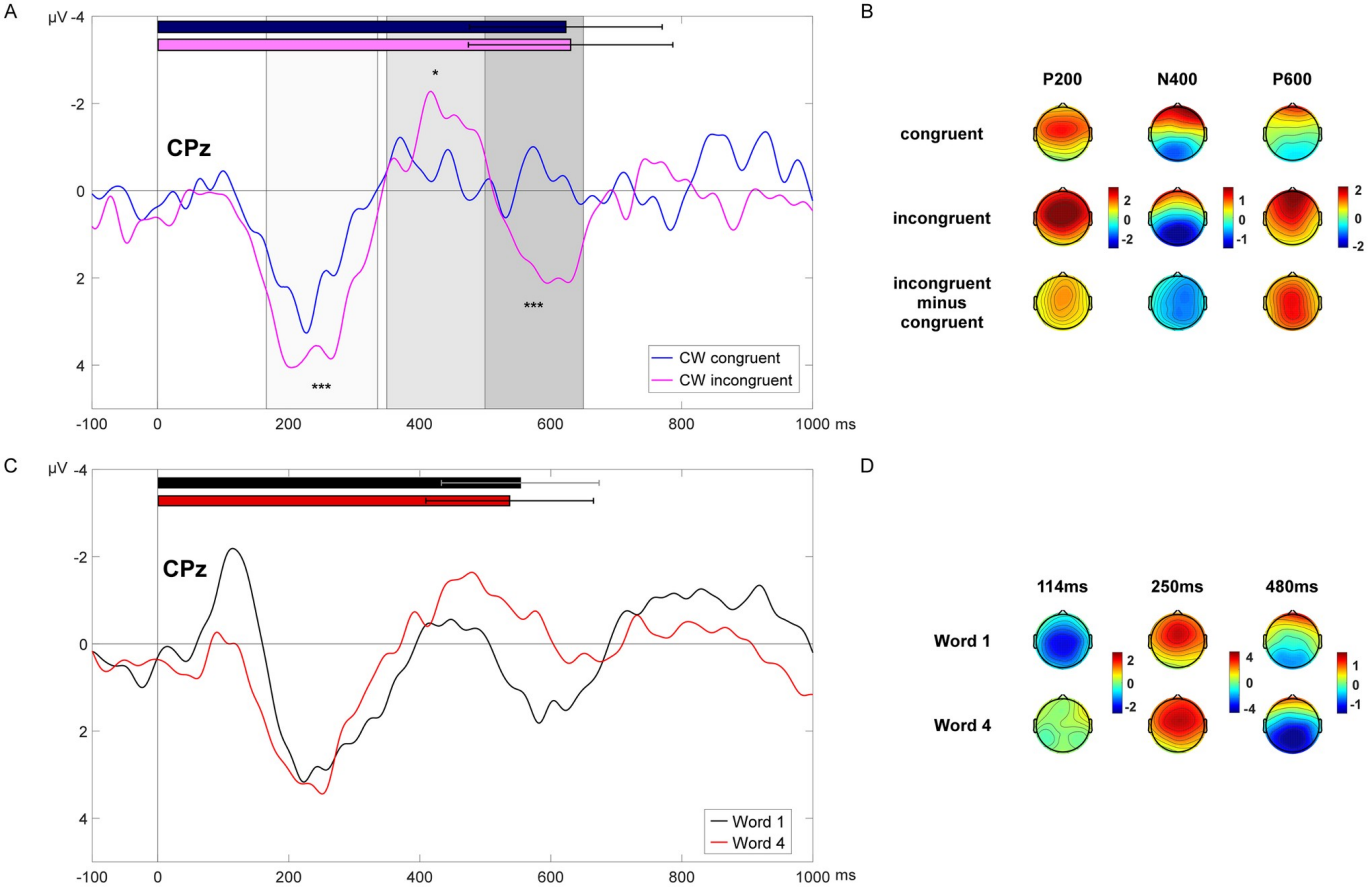
RIDE reconstructed ERP results. (A) Grand average RIDE reconstructed ERP at CPz of the critical word (CW, listened word 8) for the congruent (blue line) and incongruent condition (magenta line); on the same time scale the average speech times of the CW congruent (blue bar) and CW incongruent (magenta bar) are shown with *SD*. Time-windows for statistical analysis are depicted in gray shades for the P200 (light gray), the N400 (middle gray), and the P600 (dark gray). Significant effects of congruency on the amplitude are marked with asterisks. * significant at *p* < 0.05; *** significant at *p* ≤ 0.001. (B) Grand average mean topographies of the P200 from 166–336 ms (first column), the N400 from 350–500 ms (second column), and the P600 from 500–650 ms (third column) for the CW congruent (upper row), CW incongruent (middle row), and CW difference (incongruent minus congruent; bottom row). (C) Grand average RIDE reconstructed ERP at CPz of listened word 1 (black line) and listened word 4 (red line); on the same time scale the average speech times of word 1 (black bar) and word 4 (red bar) are shown with *SD*. (D) Grand average peak topographies of the N100 at 114 ms, P200 at 250 ms, and N400 at 480 ms for word 1 and word 4.

For the critical word (CW), the RIDE reconstructed ERP showed an expected time course for this type of stimulus material: a positive peak around 200 ms, a negative peak around 400 ms, and a positive peak around 600 ms (see Fig 3A). To test the influence of congruency (congruent vs. incongruent) on the observed ERP components, two separate 2 x 4 repeated measures ANOVA, midline (four level factor electrode along the midline) and lateral (four level factor quadrant along sites), were computed for each component (P200, N400, P600).

The mean amplitude of the P200 (166–336 ms) was significantly higher for the incongruent compared to the congruent CWs [midline: *F*(1,25) = 17.17, *p* < .001, *η*_*p*_^2^ = 0.407; lateral: *F*(1,25) = 15.76, *p* = .001, *η*_*p*_^2^ = 0.387]. Visual inspection of the grand average topography suggests the strongest P200 among frontal to medial sites (compare Fig 3B). However, there was no significant interaction between congruency and electrode or congruency and quadrant [midline: *F*(3,75) = 0.77, *p* = 0.452; lateral: *F*(3,75) = 0.45, *p* = 0.65].

The mean amplitude of the N400 (350–500 ms) was significantly lower (more negative) for the incongruent compared to the congruent CWs [midline: *F*(1,25) = 4.69, *p* = .04, *η*_*p*_^2^ = 0.158; lateral: *F*(1,25) = 4.63, *p* = .041, *η*_*p*_^2^ = 0.156]. There was no significant interaction between congruency and electrode or congruency and quadrant [midline: *F*(3,75) = 0.08, *p* = 0.86; lateral: *F*(3,75) = 0.99, *p* = 0.373].

For the P600 (500–650 ms), congruency had a statistically significant effect on the amplitude [midline: *F*(1,25) = 16.82, *p* < .001, *η*_*p*_^2^ = 0.402; lateral: *F*(1,25) = 15.53, *p* = .001, *η*_*p*_^2^ = 0.383], with higher amplitudes for the incongruent compared to the congruent condition. There was no significant interaction between congruency and electrode or congruency and quadrant [midline: *F*(3,75) = 0.77, *p* = 0.437; lateral: *F*(3,75) = 0.57, *p* = 0.534].

The RIDE reconstructed ERP of the 1^st^ word of each sentence (i.e., the ERP in response to the other participant saying the first word) and 4^th^ word of each sentence (i.e., the ERP in response to the other participant saying the fourth word) are shown in Fig 3C. The ERP for the 1^st^ word shows a negative peak at around 114 ms. The latency and the corresponding scalp topography suggests that this is an auditory N100 in response to the speech onset of the first word of each trial. The amplitude of the N100 is significantly reduced from 60 to 160 ms for the succeeding words, as indicated by a significant effect of word position (1^st^, 2^nd^, 3^rd^, and 4^th^) on the N100 amplitude [midline: *F*(3,75) = 13.97, *p* < .001, *η*_*p*_^2^ = 0.359].

## Discussion

This study is part of a larger research project in which we target neural correlates of social interactions, with a focus on verbal interactions. Within the endeavor to bring EEG outside the lab [23,53,54], we employ a wireless EEG system in our research line. Our intention is to study the neural processes underlying social interactions in naturalistic scenarios with as little constraints as possible. We approach this goal step by step. Our first study showed that established linguistic components (e.g., the N400 effect) could be measured when introducing a turn take on the critical word [17]. In the present study, we took the next step and implemented a scripted word-by-word interaction: two persons had to read aloud word-by-word sentences, where the semantic expectation for the sentence continuation was manipulated. We predicted to see established linguistic ERP effects to expectancy modulations and differences in speech timing after unexpected compared to expected events despite the participant’s active speech production.

We could show that the word-by-word task gives a variety of behavioral measures, e.g. for utterance timing from word to word, with a distinct pattern per sentence. The ERP results showed the predicted electrophysiological response to linguistic stimuli: an N100 for the first spoken word of a sentence and a P200—N400—P600 effect for the critical eighth word. Each of the ERP components for the critical word was modulated significantly by the semantic expectancy of the word. Unexpected words enhanced the ERP component response, with more positive amplitudes for the P200 and P600, and more negative amplitudes for the N400. These results pave the way for the next step: to allow an unscripted word-by-word interaction between participants while assessing the underlying neural and behavioral patterns.

We have shown that the word-by-word paradigm allows assessing speech times of utterances and the reaction times to speak during the interaction. By preventing coarticulation [55] from word to word due to the constant alternation between speakers, the word-by-word turn-taking allows to have major control of the utterance timing. For the present paradigm, the average RTs over the experiment were similar between interacting pairs. Our question here was, if congruency of the word would have an effect on the RTs to speak, where we expected an increase in reading aloud times for incongruent compared to congruent words. This hypothesis was based on observations of the game and self-paced reading studies, which have shown an increase in reaction time (to click) for incongruent words and their follow ups in sentences (e.g., [6]). Seeing increases in reaction times (to speak) in the present paradigm would point to the fact that the processing of the incongruent word takes longer, enhancing the needed time to produce the word and/or the following words. Due to the turn-taking nature of the paradigm, we included the word position as a co-factor in the analysis. We will discuss in the following paragraphs the findings for the sequence CW+1, CW+3, CW+5 spoken consecutively by one participant and the word-sequence CW, CW+2, CW+4 spoken consecutively by the other participant.

In contrast to the predicted increase in reading aloud times for unexpected words, we found a significant decrease in RTs for unexpected versus expected sentence continuations for the sequence CW+1, CW+3, CW+5. Findings of self-paced reading studies [6,19] suggest that an incongruent word would lead to enhanced reaction times. However, a number of points are different between this setup and self-paced reading setups: in silent reading studies (1) semantic violations lead to increased reading times also for sentence final words, which was not the case here, (2) there is often a plausibility judgement task after the sentence, pointing to a more explicit semantic analysis of the sentence, which was also not incorporated here, and (3) the setup usually asks the participant to read a sentence word-by-word by self-paced clicking for the next word to display, whereas our setup demanded the participant to read aloud leading to activations different from clicking. The high cognitive load of the task (reading aloud, taking turns for each word, staying focused throughout the prime and 13 words of a sentence, moving as little as possible) may have shadowed the expected effect. In addition, the violation was induced by a prime at the beginning of the sentence rendering the sentence ambiguous but not semantically implausible after reanalysis. A possible explanation of the result that participants were significantly faster in reading aloud words after unexpected compared to expected CWs, could be a reallocation of attentional resources [56]. Specifically, vigilance might be increased by a context that is less predictable and, hence, unexpected [57], leading here to a smaller RT to read aloud the word following the unexpected heard CW. We expect our follow-up study to be able to shed some light on these results.

For the sequence CW, CW+2, CW+4, we did not find a RT congruency effect in either direction. An explanation might be the fact that for one sequence the unexpected word was produced (CW, CW+2, CW+4), whereas in the other condition (CW+1, CW+3, CW+5) it was perceived. In line with our prior argument of an increased vigilance after a heard CW, the difference of perceiving and producing the CW could have an impact on the processing speed of the word in question. The next RT in this sequence is measured on the CW+2, where such an increased vigilance effect is possibly over. However, this effect is hard to disentangle here, since participants must quickly react to the ongoing sequence, listen and read aloud the next words. Moreover, there is a difference in trial numbers (expected vs. unexpected) between sequences CW, CW+2, CW+4 (100 expected spoken words vs. 20 unexpected spoken words) and CW+1, CW+3, CW+5 (80 expected spoken words vs. 40 unexpected spoken words). The arguments of dissimilarity between self-paced reading studies and the present study (semantic ambiguity vs. anomaly and implicit vs. explicit measure) hold of course for this sequence too. However, we assume the cognitive load and the difference in trial numbers to be the main reasons for not finding significant congruency effects for this sequence.

On the neural level, we found meaningful ERPs as a response to the listened utterances in this word-by-word setting. In particular, we found a significant auditory N100 response to the first spoken word in a sentence, which significantly decreased for the following words. For the critical word, we found established linguistic ERPs: a P200, N400, and P600 response, all of which were modulated by the semantic violation of the word in the sentence context.

Similar to previous findings [17,18,58], the P200 ERP showed more positive amplitudes for unexpected sentence continuations compared to expected ones. Visual inspection of the grand average topography suggests the strongest P200 among frontal to medial sites, which is in line with previous results (compare above). The role of the P200 for semantic analysis is still debated. We follow the interpretation that the P200 indexes the comparison of an (auditory) input with an internal representation or expectation in memory or language context [59,60]. The violation of this (auditory) expectation leads to processing costs reflected in the P200 response.

The N400 ERP effect is well known for its link to semantic expectation and analysis (for a review see Kutas & Federmeier, 2011). In contrast to the auditory expectation violation reflected in the P200 response, the N400 effect is linked to the processing of the semantic concept of the expected word [27,61]. Note that it is still discussed if the N400 is rather an index of semantic expectation (i.e., prediction) or semantic integration (compare [10,13,28]). Furthermore, N400 effects have also been observed for pictures and non-linguistic material (e.g., [62,63]), therefore relating it to a more general memory access. We interpret the N400 effect here as showing that expectations on sentence continuations are built during a word-by-word game, which lead to a predicted neural response when violated. Further, it shows that meaningful language-related ERPs can be measured in this interactive setup. Participants processed the given prime at the beginning of a trial and used it to fasten semantic analysis during the turn-taking. This means that future utterances of the dialogue partner are predicted similarly in a word-by-word scenario as in a dialogue (i.e., sentence) scenario. Topographically, the N400 was strongest along medial to posterior sites, which is expected for this type of linguistic material ([17,64–66], for a review see [10]).

The P600 ERP points to a reanalysis of the linguistic material and is usually linked to syntactic violations [11]. Studies focusing on the N400 effect, however, often report a P600 modulation or so-called late positivity [14]. The P600 response in these studies is seen as a reanalysis of the whole sentence structure, regardless of the presence of syntactic violations [67]. Lately, the presence of the P600 is also being discussed for semantic anomalies [68,69], reinforcing the role of the P600 as an index of reanalysis. The present word-by-word setup also leads to the necessity of reanalysis of the sentence. Even more so, when accounting for the unexpectedly primed sentence continuations. The critical word is not semantically anomalous, but it is semantically unexpected and still plausible for the other meaning of the presented homonym in the sentence. Thus, though not predicted, the present P600 modulation (i.e., more positive for unexpected words) for our linguistic stimuli is a meaningful ERP response.

For all ERP components analyzed in this study, i.e., the P200, N400, and P600, no significant interaction between the congruency effect and the site of the effect was found. Possibly, the dense centro-parietal coverage of EEG electrodes in contrast to the less dense coverage over frontal sites could play a role in (not) capturing such differences between regions. However, the absence of a statistically significant interaction between congruency and site of the effect does not rule out that the neural generator of the component is located at a specific site [70,71].

The question remains on how to interpret the different findings on the neural and behavioral level. We confirmed the predicted effect of semantic violations leading to increased P200 and N400 amplitudes. However, the behavioral measures provide a less clear picture on the effect of semantic violations on the RT to speak. The lack of a significant effect for the sequence CW, CW+2, CW+4 and the inverse effect for sequence CW+1, CW+3, CW+5 (i.e., decreased RTs to speak for unexpected words) could also suggest that RT measures cannot capture the underlying process reliably (at least not with the present material). Whereas, at the same time, EEG is able to measure the cost of encountering unexpected words in a sentence. Even though this seems to be the case in the present study, a combination of both measures, RTs and ERPs, might provide the best framework to understand the core processes at and after encountering an unexpected linguistic input.

Ostensibly, this paradigm with its scripted interaction is still quite different from the open-ended interaction that can be seen on an improvisational theater stage. That is, the interaction on the stage is more dynamic, chaotic, creative, unpredictable and funny, than the paradigm we have used here. However, as such, the open stage scenario is not suitable to study the build-up of expectations using current EEG technology. For example, it cannot be assured that a sufficient number of “unexpected” events will occur that would allow for an ERP analysis. Furthermore, it is impossible to assess which utterances of one person should be considered unexpected for the other person. Finally, it often occurs on stage that the confusion is so large that no coherent sentences are uttered at all.

Our paradigm, despite being scripted, already incorporates many aspects of open interactions: participants both speak and listen, and they have to coordinate with their partner in taking turns. As such it allows studying some key components of interaction in natural environments [72], while still having sufficient experimental control. We now have a paradigm, the technical setup as well as the methodological tools to study the effect of improvisational theater training on social interaction [73]. Specifically, we can now focus on the role of expectation building and expectation adjustment while interacting. It is possible to measure behavioral and neurophysiological underpinnings of these interactions, relating them to the expected or unexpected utterances.

Obviously, several constraints of the current paradigm need to be loosened to study interaction in its more natural form. Instead of reading the sentences, the participants have to generate the sentences themselves. It then has to be made sure that sufficiently many unexpected words occur. This could be done, for example, by having a confederate that introduces unexpected words into the conversation at well-defined moments. A second possibility is to ask participants to describe jointly a picture, but present different pictures to each participant.

## Conclusion

The word-by-word paradigm was used here as a controlled approach of measuring ERPs in a dynamic setup. Engaging two participants in a scripted interaction allowed us to retain a high degree of experimental control and to test thereby the feasibility of measuring ERPs in such a dynamic setup with variations in the auditory input. We assessed utterance timing along with RTs, and saw meaningful and expected ERP responses to expectancy violations. We conclude that the word-by-word paradigm allows to measure neural correlates of such a verbal interaction. Based on these results, the next step must be to facilitate own language production while retaining sufficient experimental control for the analysis of the EEG data. A further improvement of the paradigm will be to reduce the cognitive load of the task, i.e., that the participants can focus more on the semantic analysis and the production of the utterances. The common concept of these two points—own language production and decreased cognitive load—is to increase the engagement of the participant, leading to a more open and natural word-by-word game while preserving experimental control.

Our research can be embedded in the movement of a social neuroscience: the current efforts of moving towards more natural study setups, using more natural stimulus material, and introducing interaction between participants. It is far from trivial to combine open speech production during interaction with sufficient experimental control to analyze EEG data meaningfully. The word-by-word paradigm can be used to study such neurophysiological responses to expectation violations during verbal interaction. Aside from expectation building, the word-by-word paradigm can be further used to study many interactional aspects, such as conceptual pacts [74,75] or behavioral and neural synchronizations [16,76,77] during interactions. In conclusion, the word-by-word paradigm provides an experimental framework to study social interaction.

## Supporting information

S1 File Paradigm. (DOCX)

S1 Table Paradigm trial overview. Trials in which the critical word (CW) was read aloud by the participant are shown in brackets. Congruency is defined by the prime at the start of each trial, which may differ for each participant. One trial includes the presentation of the prime and reading aloud word-by-word two sentences with 13 words in total. The spoken word sequence for each participant is shown. For example, participant 1 listens to the CW and reads aloud words CW+1, CW+3, and CW+5, while participant 2 reads aloud words CW, CW+2, and CW+4 in the same trial. Trials used for EEG analysis (per participant) are highlighted in bold. All spoken words were used for the RT analysis, split into word sequences CW+1, CW+3, CW+5 or CW, CW+2, CW+4.
(DOCX)

S2 Table Trial type stimuli examples. An example is shown for each participant, each congruency condition, and each listening/speaking the 8th word condition. The respective condition is the title of each subbox (EEG / Non-EEG Trial = used / not used for EEG analysis, CW was listened / spoken; Participant 1/2 = condition for this participant; congruent/incongruent = Prime shown at the beginning of the trial was congruent/incongruent to the CW). The two possible primes of each sentence are shown at the beginning, where the colors (blue or green) refer to the congruent or incongruent 8th word option. The homonym is highlighted in orange. The action of each participant is described below the respective trial word (read silently the prime, speak or listen to the word of the sentence).
(DOCX)

S3 Table GLMM results. GLMM was fitted with a Gamma probability distribution and an inverse link function. Coefficients and SE are NOT backtransformed. The predicted RT is the backtransformed RT value in seconds: intercept is the overall mean predicted RT, mean predicted RT for congruency is for moving from congruent to incongruent condition (all other factors remaining constant), and mean predicted RT for word position is for moving from the first word position to the last word position (all other factors remaining constant).
(DOCX)
