# Supplementary material for: Studying brain activity during word-by-word interactions using wireless EEG
Source: PLoS One. 2020 Mar 24;15(3):e0230280. doi: 10.1371/journal.pone.0230280 (PMC7092963; doi:10.1371/journal.pone.0230280)
Supplement: S1 File — (DOCX) [file pone.0230280.s001.docx]

**Supporting information**

**Paradigm**

**Trial construction**

In this paradigm (see setup in S1 Fig), EEG and audio was always recorded for a pair of two participants that interacted with each other. The utterances of one participant were used as (heard) stimuli for the other participant. Three major criteria were considered for the construction of the language material (compare [1]): (1) a minimum of 40 trials in which each participant listened to the unexpected CW, (2) a balance of 25% incongruent trials to 75% congruent trials for each participant, and (3) a subset of trials where the CW was read aloud and incongruent was needed for each participant to address concerns of different congruency expectation for the CW in trials, where the participant listened to the CW versus read it out. These criteria lead to 240 experimental trials in total shown in S1 Table. An example for each trial type and task of each participant is given in S2 Table.


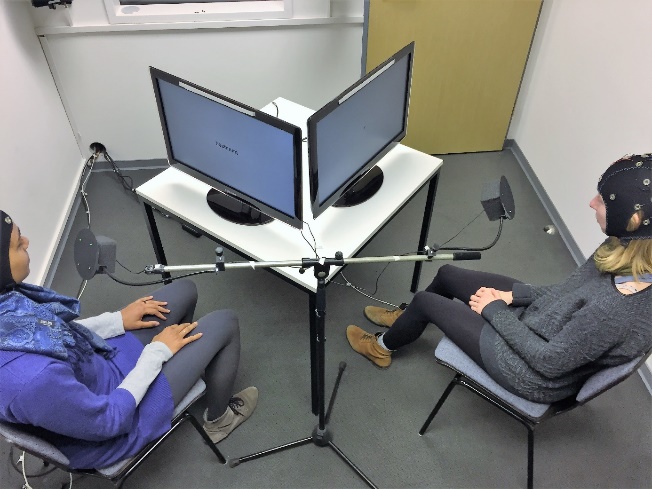


**S1 Fig.** **Experiment setup, where participants sat next to each other.** The setup included two displays, one in front of each participant. Either one was not able to see the display of the other. A tripod with two extensions was located between them, where on each extension a microphone (i.e., a split up stereo microphone) with audio pop shield was attached. While one participant saw and read aloud a word of the sentence, the other participant saw a fixation cross.

**Linguistic material**

Each trial of the word-by-word paradigm consisted of 13 words, where the 4^th^ word was always the homonym and the 8^th^ word was always the ambiguity dissolving critical word. Sentences were constructed such that both meanings of the homonym were plausible and acceptable until the CW. The sentences’ plausibility was ranked by four German native speakers on a scale from 0 (not plausible at all) to 4 (very plausible). For the plausibility rating, each sentence was presented with the congruent prime. The average plausibility was 3.22.

The first word of a trial was always a male (50%) or female (50%) forename. The second word was always a verb. Verbs were selected in such a way that we avoided common co-occurrences of one meaning of the homonym and the verb. In most cases (~ 75%), the third word was a determiner. In cases where this was not possible (e.g., when the homonym differed in article dependent on its meaning), prepositions, pronouns, adjectives, adverbs or numerals were used as third words. The fourth word was always the homonym. The fifth word was always a personal pronoun, with two exceptions (an adverb and a preposition), where no other sentence construction was possible. The sixth word was always a verb. The seventh word was, in most cases (75%), a determiner. Otherwise, the seventh word was a personal pronoun, a preposition, a pronoun, a numeral, a conjunction or an adjective. Each experimental trial had two possible eighth words (with three exceptions – filler trials – with only one eighth word, where no other sentence construction was possible, i.e., 477 words in total): one eighth word for one meaning of the homonym and one eighth word for another meaning of the homonym (partly adapted from the pretested set of [2]). The ninth word was a conjunction, a relative pronoun, an interrogative pronoun, a preposition or an adverb. Words ten to thirteen were variable.

Two noun primes were selected for each experimental trial (i.e., 480 words in total, partly adapted from the pretested set of [2]). One prime for one meaning of the homonym, matching one of the eighth words, and a second prime for a second meaning of the homonym, matching the second eighth word (see examples in S2 Table). Whenever possible, primes were kept short and common words were selected. We avoided exceptionally low-frequent words.

**Evaluation results**

After the word-by-word experiment, participants were asked to fill out an evaluation questionnaire. On a five-point Likert scale (0=‘not at all’,1= ‘a bit’,2= ‘neutral’, 3=‘somewhat’, 4=‘very’) they had to rate (among others) how natural the interaction was, how pleasant the interaction was, and how pleasant the interaction partner was. For this, the participants were seated in two separate rooms to prevent socially expected answers.

To test if the ratings within a pair depended on the partners rating, a permutation test with 100000 iterations was calculated. The difference value (rating from one participant subtracted by the rating of his partner) was calculated, squared and summed up for all pairs. The sum was divided by the number of pairs and square rooted, leading to an observed value. A distribution of values was calculated by randomly assigning participants to a pair.

We evaluated the ratings for the feeling of naturalness of the interaction (*Mdn* = 2), the pleasantness of the interaction (*Mdn* = 3) and the pleasantness of the interaction partner (*Mdn* = 4). Results for the subjective feeling of naturalness of the interaction indicate a significant co-dependency of ratings within a pair (*Mdn* = 2, *Y* = 1.3, *p* = .011). The ratings of the pleasantness of the interaction were not co-dependent within a pair (*Mdn* = 1.71, *Y* = 1.62, *p* = .308), whereas the ratings of a pair were co-dependent for the experienced pleasantness of the partner (*Mdn* = 1.3, *Y* = 0.88, *p* = .019).

In view of the experienced naturalness of the word-by-word paradigm, there is room for improvement. The evaluation displayed a medium rating for naturalness on average with a broad distribution of answers. Interestingly, the rating of one participant of a pair depended on the partners rating, meaning that the partners mutually influence each other’s feeling of naturalness. This was also the case for the experienced pleasantness of the interaction partner. We deduce that a pleasant and naturalistic word-by-word game relies on the sympathy between the interacting partners.

**Package loss correction**

EEG sets in the present experiment were recorded from two persons simultaneously using two wireless EEG systems ([www.mbraintrain.com](http://www.mbraintrain.com)) with a sampling rate of 500 Hz. The EEG signal during measurement is stored in small chunks and send wirelessly via Bluetooth from the amplifier to a USB dongle attached near the participant. The USB dongle is connected by wire to the respective recording computer. The data was collected in 16 blocks. The immediate proximity of the two wireless EEG systems and the relatively high sampling rate can lead to sporadic wireless signal overlays resulting in a package loss of one participants’ EEG recording. The recording system corrects for these losses by interpolating the previous sample. New incoming samples are then delayed by minor time segments. This effect is identifiable by calculating the real sampling rate during the measurement. If it falls below 500 Hz, it points to the described problem. Moreover, it can be visualized in a channel ERP image for e.g. visual responses at channel O1. Both, sampling rate calculation and visual ERP analysis (see S2 Fig – *before* package loss correction), suggested some package loss during measurement for participants 2 to 5, 7 to 13, 15 to 17, 20 and 21, as well as participant 25. Since the measured signal is minimally delayed, a possibility for repair is to estimate the real latency of the visual ERP for each trial and correct the EEG triggers according to the measured lag between current and actual onset of the visual response.

Actual latencies of the visual ERP were estimated in the ASR preprocessed EEG data (see section 2.7 in the main text for details on this preprocessing package). Data for each participant were epoched to the visual presentation of a word on the screen between -500 ms and 1500 ms. Epochs were baseline corrected from -100 to 0 ms. To estimate the actual latency of the visual EEG response for each trial, we applied the woody function incorporated in the RIDE toolbox [3,4]. Since the woody function is a pre-step for latency estimation in the RIDE toolbox, it is a sensitive measure for the real onset of a stimulus response, in our case the visual stimulus response. Visual inspection of the calculated lags between current and actual visual ERP onset suggested that the method might be overly sensitive for our purpose. Therefore, we applied a filter (20^th^ order one-dimensional median filter in Matlab R2016a) to the calculated lags of each participant and each trial.

The new visual response lags for each trial were now used to correct the EEG sets with package loss (participants 2 to 5, 7 to 13, 15 to 17, 20, 21, and 25). The calculated lag for each trial was subtracted/added to the visual presentation EEG triggers and the five consecutive EEG triggers to it. These five consecutive triggers included the speech onset of the presented word, the speech offset of the presented word, the visual presentation of the next word to the other participant, the speech onset of the presented word of the other participant, and the speech offset of the presented word of the other participant. Since the calculation of lags was based only on visual stimuli responses, the correction was independent of the EEG segment of prime interest: the listened CW spoken by the other participant. The corrected sets’ visual response channel ERP image of O1 points to a successful package loss correction in aligning the visual response peaks (see S2 Fig – *after* package loss correction). Further, the correction was quantified comparing the amplitude of the single participant average visual ERP before and after package loss correction. All participants showed an increase of mean visual ERP amplitude after correction.


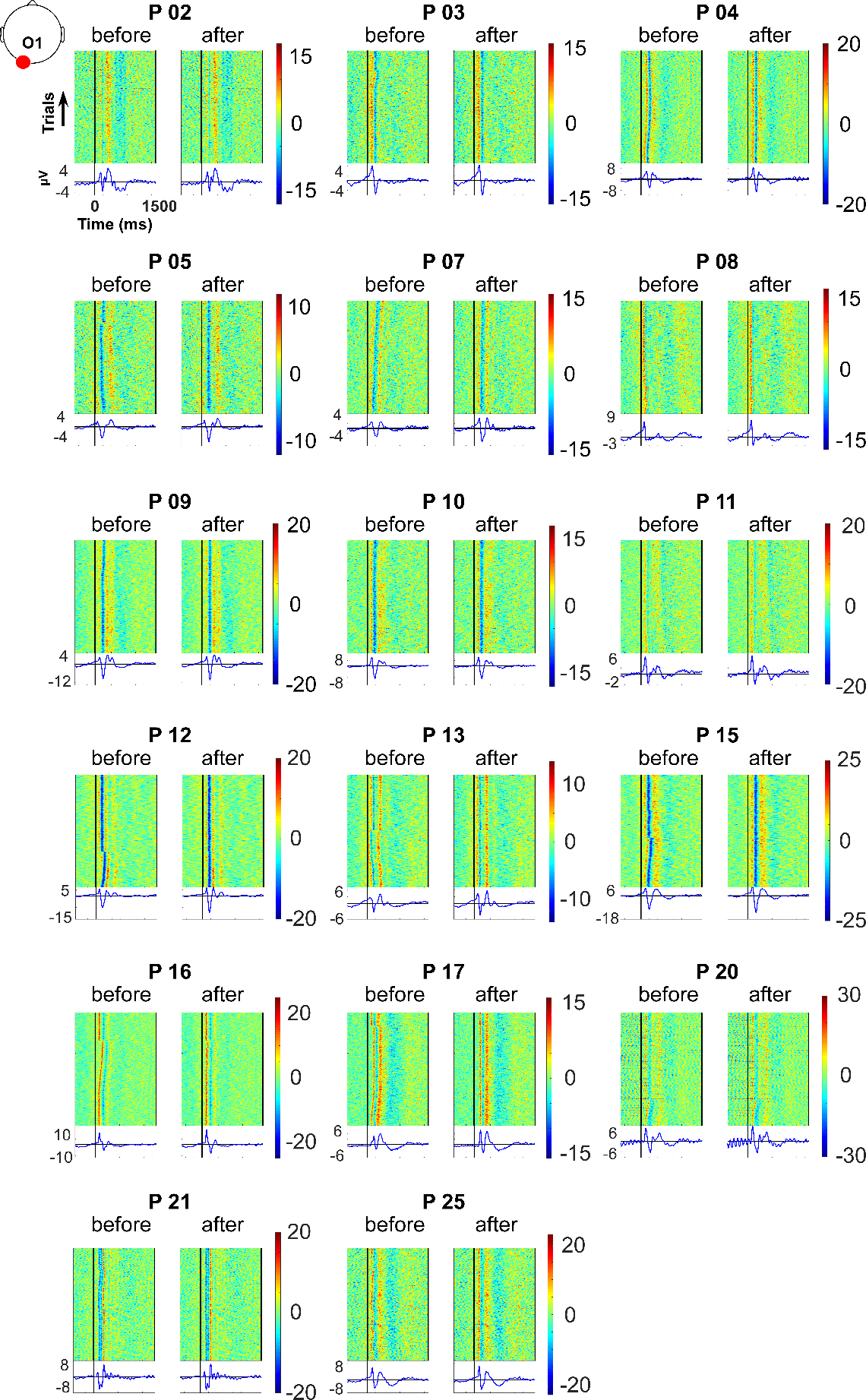


**S2 Fig.** **Channel ERP image at O1 for each ‘corrected’ participant ‘before’ and ‘after’ package loss correction.** Participant number is depicted above each image. Zero-point is the onset of the visual stimulus (word on the screen). The visual ERP is shown at the lower part of each image with respective scales on the left. The upper part of each image shows the EEG activity for each trial (from bottom to top) with respective power scales on the right hand side. Data was collected in 16 blocks with restart of the system after each pause (see shifts in ‘before’ plots for specific trials of one block). The shifts are straightened ‘after’ correction for sample losses. The visual ERP response is also mostly enhanced ‘after’ correction.

**ERP data**

An overview of the different modality conditions, i.e., heard, spoken, and visual CW, before and after RIDE application is given in S3 Fig.

To allow for comparison, we show the ‘raw’ ERP data (preprocessed as described in section 2.7) before RIDE application for the heard critical word for congruent and incongruent conditions (see S4 Fig). The same channel and condition overview is shown after RIDE application in S5 Fig. Both conditions (congruent & incongruent) have a similar signal-to noise ratio with regard to trial numbers (37 ± 1.9 for the congruent condition and 37 ± 2.6 for the incongruent condition). We specified a stimulus-locked component in a time range from 0 to 250 ms (i.e., the N100), a first cognitive component peaking between 150 and 350 ms (i.e., the P200), and a second cognitive component between 300 to 700 ms (i.e., the N400). A third cognitive component (i.e., the P600) was not specified, since we did not predict the component for this data. The same parameters were used for both conditions. No polarity is specified (nor can it be specified within RIDE) for a component and the algorithm is ignorant to the condition.

What is apparent from the contrast before and after RIDE is that the N400 and P600 are highly sensitive to jitter in the specified onsets of the stimulus (i.e., the CW). While a classical ERP analysis with this variable stimulus material can roughly capture an increased P200 for the incongruent compared to the congruent condition, the application of RIDE allows capturing the underlying N400 and P600.

To address concerns of false positive findings with RIDE, we applied RIDE on the heard homonym (Word 4) of the current setup. No congruency manipulation is present on this word and the division into congruent (named condition A) and incongruent (named condition B) is arbitrary. We randomly selected 40 trials for each pseudo-condition per participant and applied RIDE with the same parameters as for the CW (compare above) on the data. The results are visualized in S6 and S7 Figs. As expected, no obvious pattern change is apparent in the ERP for the listened homonym before and after RIDE application.


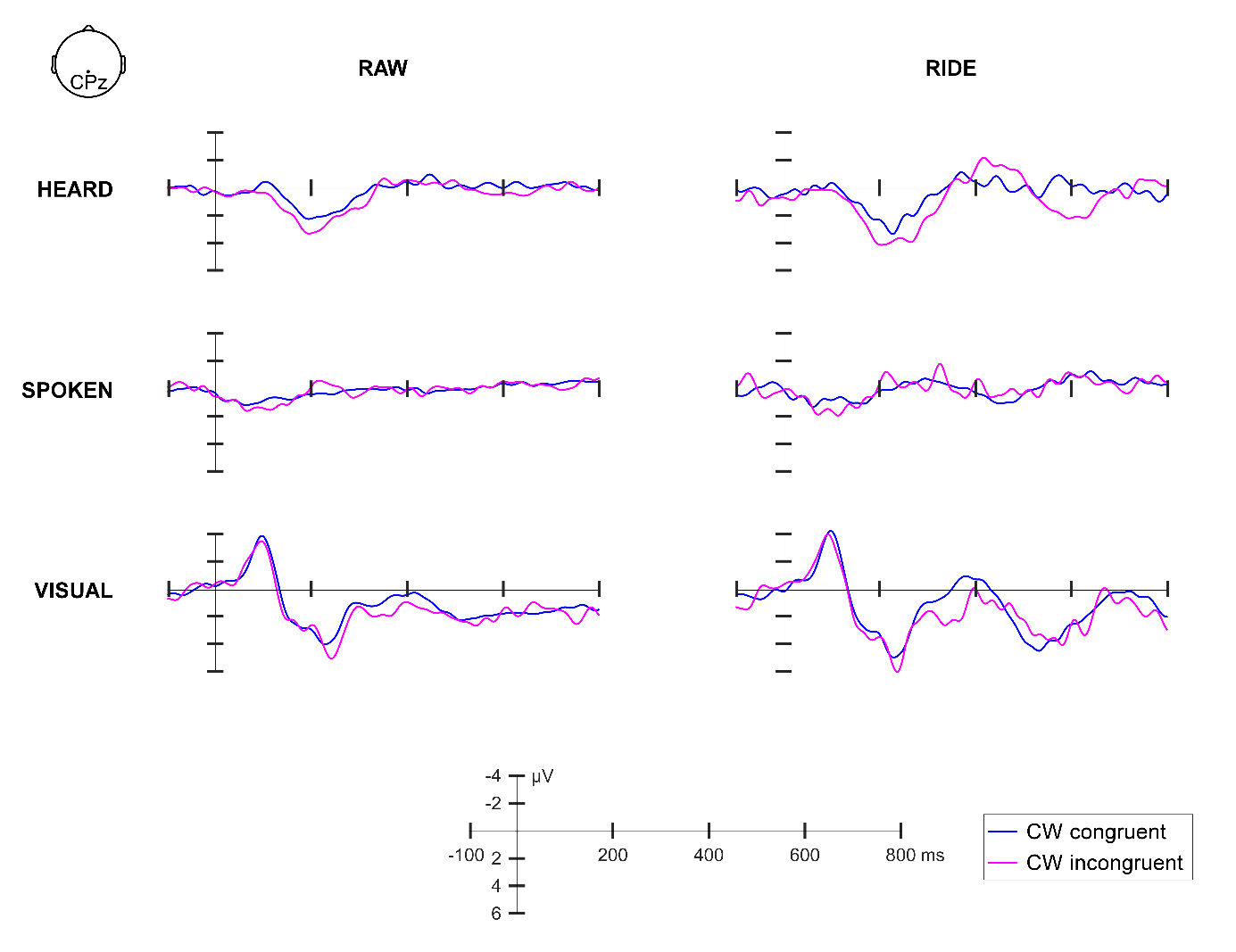


**S3 Fig. ‘Raw’ and RIDE-reconstructed grand average ERPs for congruent (blue) and incongruent (magenta) conditions at channel CPz.** Top: ERP to speech onset of the partner. Middle: ERP to own speech onset. Bottom: ERP to word on screen. The ratio of trial numbers per participant is 40 (congruent) to 40 (incongruent) for the heard condition and 100 (congruent) to 20 (incongruent) for the spoken and visual condition. Note: Congruency conditions varied for speaker and listener of the CW. Description refers to the congruency condition of the participant whose ERP is shown.


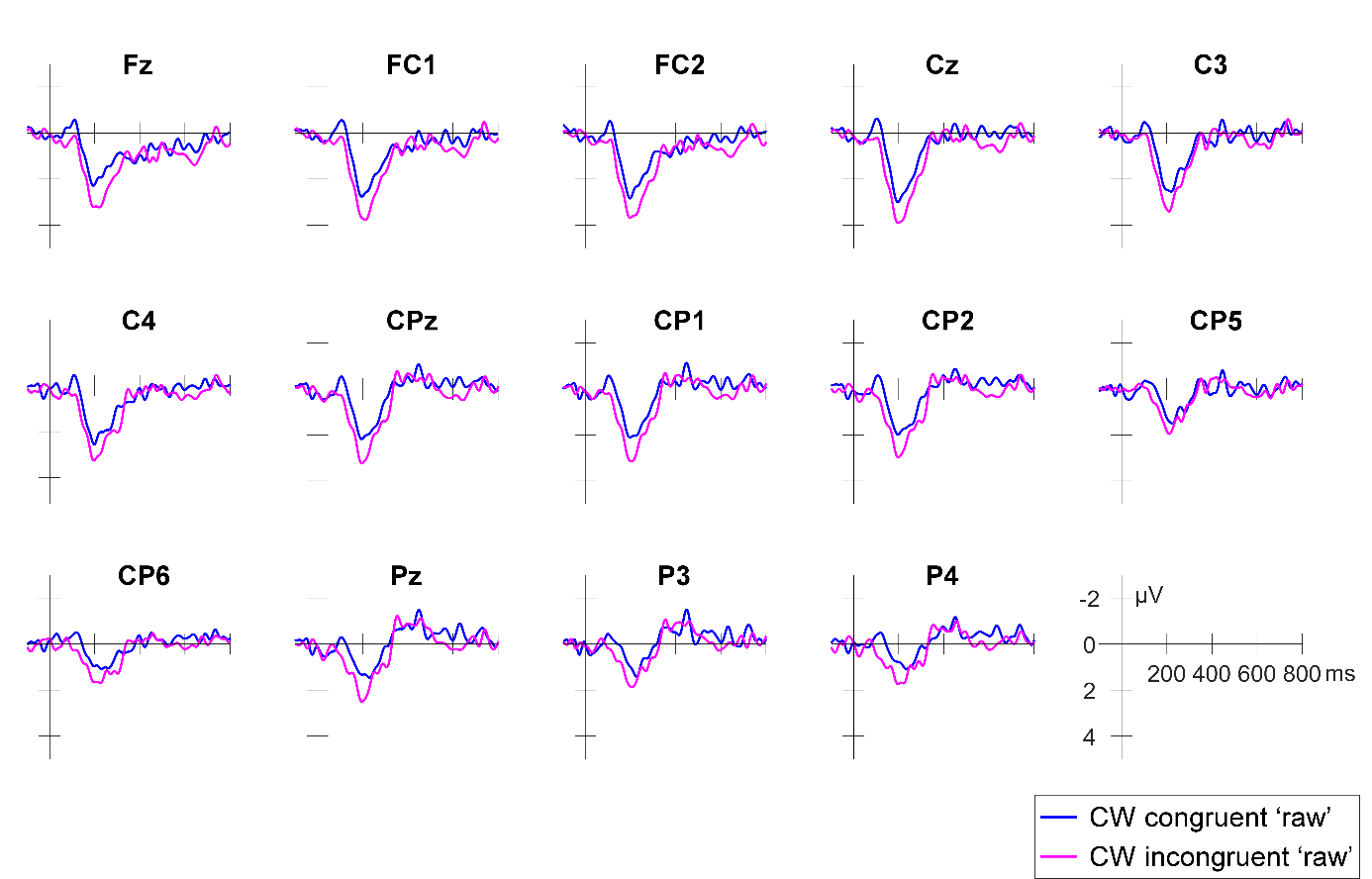


**S4 Fig.** **‘Raw’ ERPs to the onset of the critical word (CW) for congruent (blue) and incongruent (magenta) conditions.** Respective channels are indicated for each ERP. ‘Raw’ indicates before RIDE application.


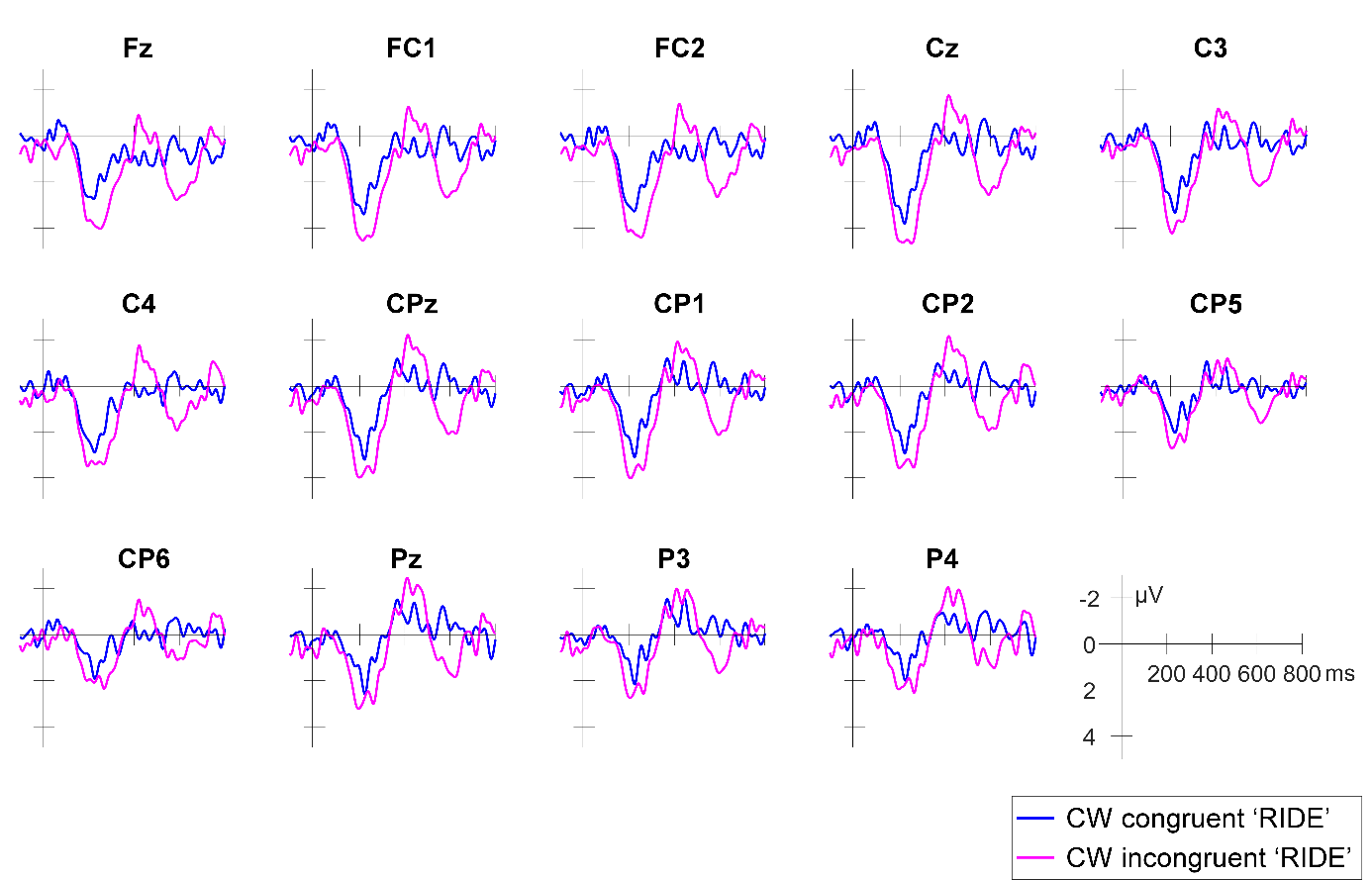


**S5 Fig. RIDE reconstructed ERPs to the onset of the critical word (CW) for congruent (blue) and incongruent (magenta) conditions.** Respective channels are indicated for each ERP.

**
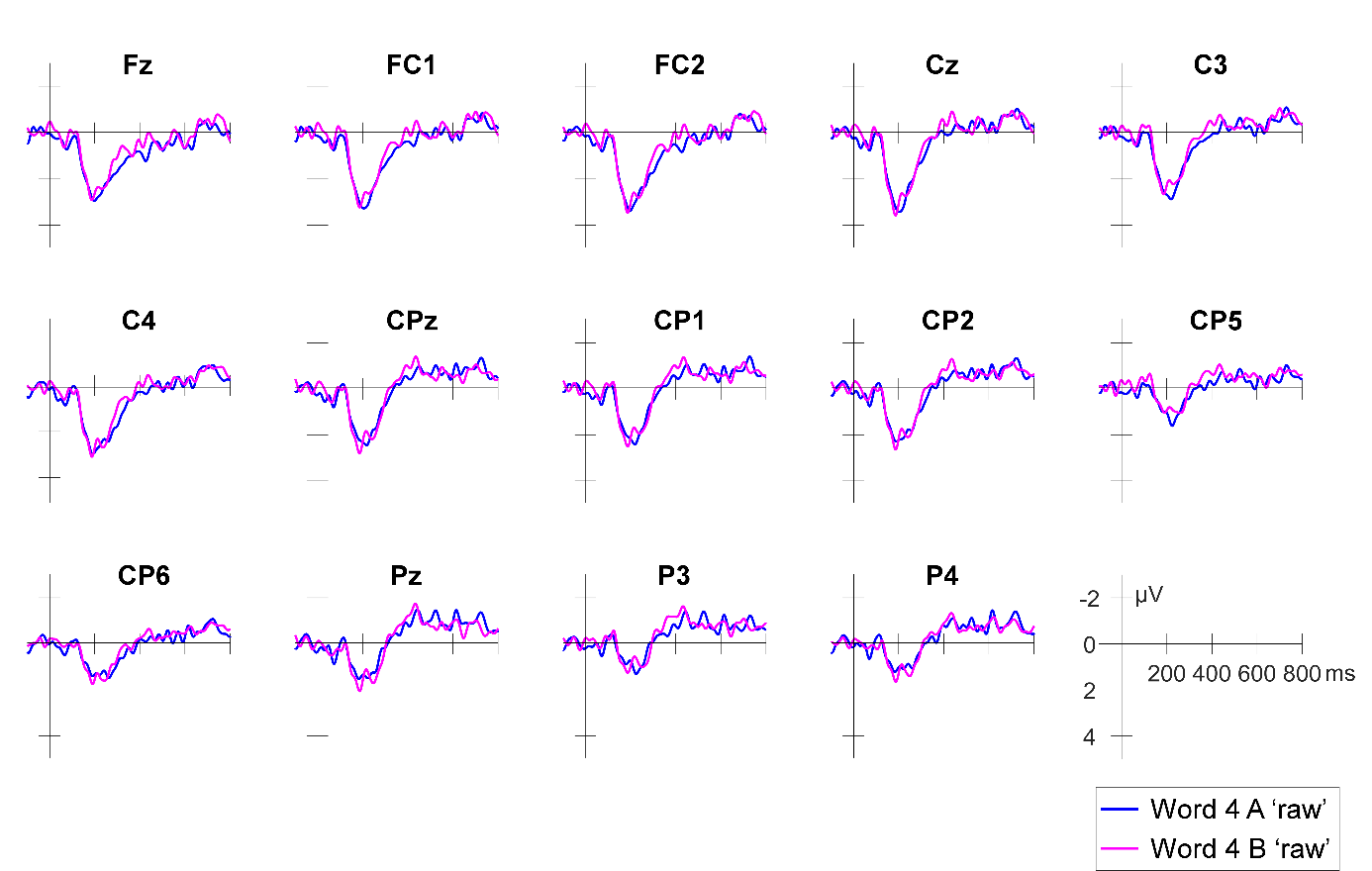
S6 Fig.** **‘Raw’ ERPs to the onset of the homonym (Word 4) for pseudo-conditions A (blue) and B (magenta).** Respective channels are indicated for each ERP. ‘Raw’ indicates before RIDE application. For the homonym, we do not expect an ERP difference between conditions.

**
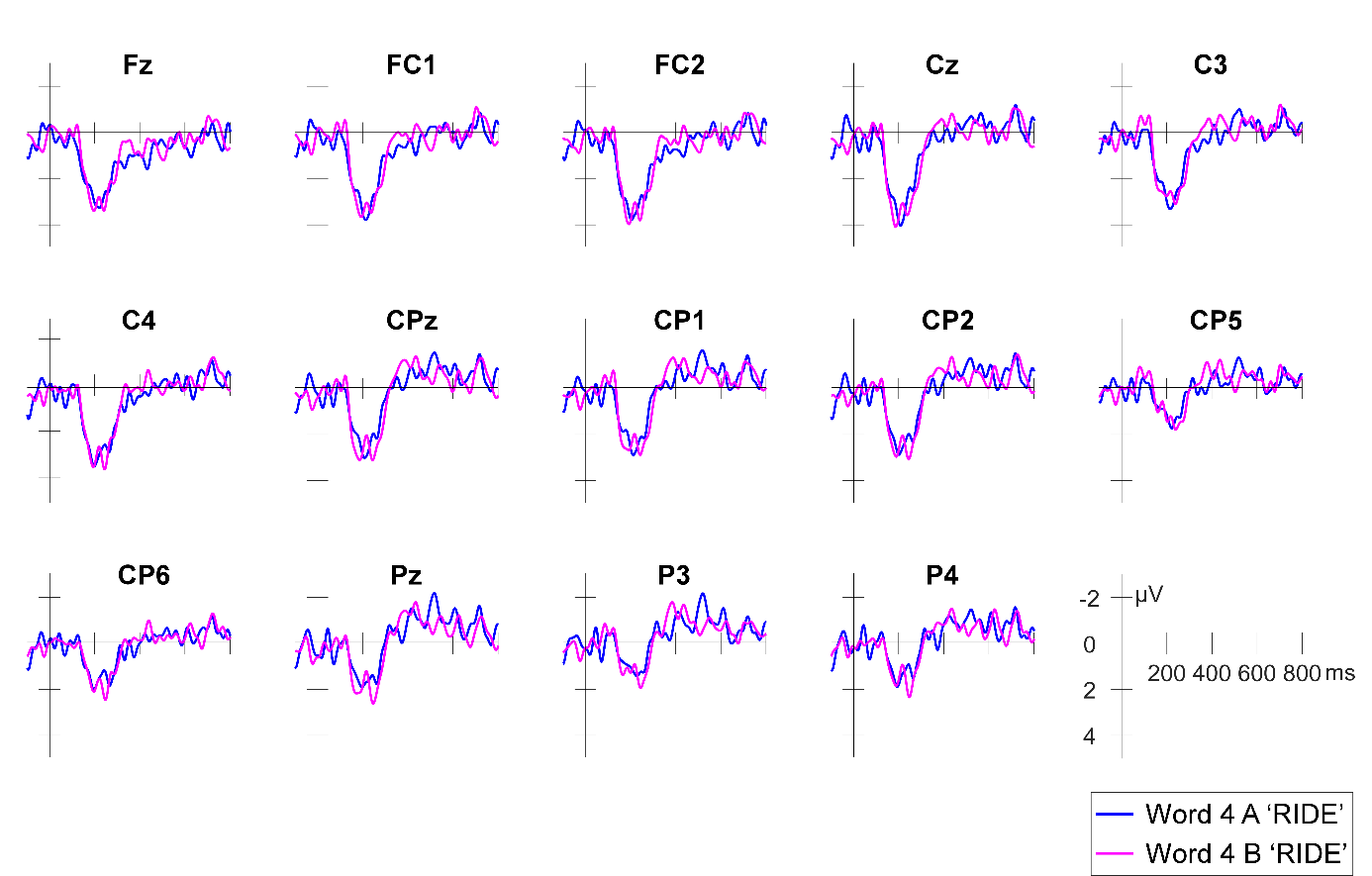
S7 Fig.** **RIDE reconstructed ERPs to the onset of the homonym (Word 4) for pseudo-conditions A (blue) and B (magenta).** Respective channels are indicated for each ERP. For the homonym, we do not expect an ERP difference between conditions, and no condition effects emerge in the RIDE reconstructed ERP.

**Supporting references**

1. Duncan CC, Barry RJ, Connolly JF, Fischer C, Michie PT, Näätänen R, et al. Event-related potentials in clinical research: Guidelines for eliciting, recording, and quantifying mismatch negativity, P300, and N400. Clin Neurophysiol. International Federation of Clinical Neurophysiology; 2009;120: 1883–1908. doi:10.1016/j.clinph.2009.07.045

2. Dshemuchadse M, Grage T, Scherbaum S. Action dynamics reveal two types of cognitive flexibility in a homonym relatedness judgment task. Front Psychol. 2015;6: 1244. doi:10.3389/fpsyg.2015.01244

3. Woody CD. Characterization of an adaptive filter for the analysis of variable latency neuroelectric signals. Med Biol Eng. 1967;5: 539–553.

4. Ouyang G, Sommer W, Zhou C. Reconstructing ERP amplitude effects after compensating for trial-to-trial latency jitter: A solution based on a novel application of residue iteration decomposition. Int J Psychophysiol. The Authors; 2016;109: 9–20. doi:10.1016/j.ijpsycho.2016.09.015
