# Supplementary material for: Studying brain activity during word-by-word interactions using wireless EEG
Source: PLoS One. 2020 Mar 24;15(3):e0230280. doi: 10.1371/journal.pone.0230280 (PMC7092963; doi:10.1371/journal.pone.0230280)
Supplement: S1 Table — Trials in which the critical word (CW) was read aloud by the participant are shown in brackets. Congruency is defined by the prime at the start of each trial, which may differ for each participant. One trial includes the presentation of the prime and reading aloud word-by-word two sentences with 13 words in total. The spoken word sequence for each participant is shown. For example, participant 1 listens to the CW and reads aloud words CW+1, CW+3, and CW+5, while participant 2 reads aloud words CW, CW+2, and CW+4 in the same trial. Trials used for EEG analysis (per participant) are highlighted in bold. All spoken words were used for the RT analysis, split into word sequences CW+1, CW+3, CW+5 or CW, CW+2, CW+4. (DOCX) [file pone.0230280.s002.docx]

**S1 Table. Paradigm Trial Overview.**

| TRIALS | ANALYSIS TYPE | PARTICIPANT 1 (P1) | | PARTICIPANT 2 (P2) | |
| --- | --- | --- | --- | --- | --- |
|  |  | CW | Spoken word sequence | CW | Spoken word sequence |
|  |  |  |  |  |  |
| **40 x** | **EEG & RT (P1)** | **incongruent** | CW+1, CW+3, CW+5 | [ congruent ] | CW, CW+2, CW+4 |
| **40 x** | **EEG & RT (P2)** | [ congruent ] | CW, CW+2, CW+4 | **incongruent** | CW+1, CW+3, CW+5 |
| **40 x** | **EEG & RT (P1)** | **congruent** | CW+1, CW+3, CW+5 | [ congruent ] | CW, CW+2, CW+4 |
| **40 x** | **EEG & RT (P2)** | [ congruent ] | CW, CW+2, CW+4 | **congruent** | CW+1, CW+3, CW+5 |
|  |  |  |  |  |  |
| 20 x | RT | congruent | CW+1, CW+3, CW+5 | [ incongruent ] | CW, CW+2, CW+4 |
| 20 x | RT | [ incongruent ] | CW, CW+2, CW+4 | congruent | CW+1, CW+3, CW+5 |
| 20 x | RT | congruent | CW+1, CW+3, CW+5 | [ congruent ] | CW, CW+2, CW+4 |
| 20 x | RT | [ congruent ] | CW, CW+2, CW+4 | congruent | CW+1, CW+3, CW+5 |
|  |  |  |  |  |  |
|  |  | Totals (*N* = 240) | | Totals (*N* = 240) | |
|  |  | 60 incongruent | | 180 congruent | |
| Totals (*N* = 240) |  | 40 x heard CWs, 20 x spoken CWs | 40 x CW+1,CW+3,CW+5, 20 x CW, CW+2, CW+4 | 80 x heard CWs, 100 x spoken CWs | 80 x CW+1, CW+3, CW+5, 100 x CW, CW+2, CW+4 |
|  |  | 180 congruent | | 60 incongruent | |
|  |  | 80 x heard CWs, 100 x spoken CWs | 80 x CW+1,CW+3,CW+5, 100 x CW, CW+2, CW+4 | 40 x heard CWs, 20 x spoken CWs | 40 x CW+1, CW+3, CW+5, 20 x CW, CW+2, CW+4 |

Trials in which the critical word (CW) was read aloud by the participant are shown in brackets. Congruency is defined by the prime at the start of each trial, which may differ for each participant. One trial includes the presentation of the prime and reading aloud word-by-word two sentences with 13 words in total. The spoken word sequence for each participant is shown. For example, participant 1 listens to the CW and reads aloud words CW+1, CW+3, and CW+5, while participant 2 reads aloud words CW, CW+2, and CW+4 in the same trial. Trials used for EEG analysis (per participant) are highlighted in bold. All spoken words were used for the RT analysis, split into word sequences CW+1, CW+3, CW+5 or CW, CW+2, CW+4.
