# Supplementary material for: Studying brain activity during word-by-word interactions using wireless EEG
Source: PLoS One. 2020 Mar 24;15(3):e0230280. doi: 10.1371/journal.pone.0230280 (PMC7092963; doi:10.1371/journal.pone.0230280)
Supplement: S2 Table — An example is shown for each participant, each congruency condition, and each listening/speaking the 8th word condition. The respective condition is the title of each subbox (EEG / Non-EEG Trial = used / not used for EEG analysis, CW was listened / spoken; Participant 1/2 = condition for this participant; congruent/incongruent = Prime shown at the beginning of the trial was congruent/incongruent to the CW). The two possible primes of each sentence are shown at the beginning, where the colors (blue or green) refer to the congruent or incongruent 8th word option. The homonym is highlighted in orange. The action of each participant is described below the respective trial word (read silently the prime, speak or listen to the word of the sentence). (DOCX) [file pone.0230280.s003.docx]

**S2 Table. Trial type stimuli examples.**

An example is shown for each participant, each congruency condition, and each listening/speaking the 8th word condition. The respective condition is the title of each subbox (EEG / Non-EEG Trial = used / not used for EEG analysis, CW was listened / spoken; Participant 1/2 = condition for this participant; congruent/incongruent = Prime shown at the beginning of the trial was congruent/incongruent to the CW). The two possible primes of each sentence are shown at the beginning, where the colors (blue or green) refer to the congruent or incongruent 8th word option. The homonym is highlighted in orange. The action of each participant is described below the respective trial word (read silently the prime, speak or listen to the word of the sentence).
