# Supplementary material for: Studying brain activity during word-by-word interactions using wireless EEG
Source: PLoS One. 2020 Mar 24;15(3):e0230280. doi: 10.1371/journal.pone.0230280 (PMC7092963; doi:10.1371/journal.pone.0230280)
Supplement: S3 Table — GLMM was fitted with a Gamma probability distribution and an inverse link function. Coefficients and SE are NOT backtransformed. The predicted RT is the backtransformed RT value in seconds: intercept is the overall mean predicted RT, mean predicted RT for congruency is for moving from congruent to incongruent condition (all other factors remaining constant), and mean predicted RT for word position is for moving from the first word position to the last word position (all other factors remaining constant). (DOCX) [file pone.0230280.s004.docx]

**S3 Table. GLMM results.**

|  | **Model summary** | | | | **Model comparison** | | |
| --- | --- | --- | --- | --- | --- | --- | --- |
| **Fixed effects** | β | SE | t-value | predicted RT | χ2 | df | p-value |
| **CW+1, CW+3, CW+5** |  |  |  |  |  |  |  |
| Intercept | 1.411 | 0.077 | 18.33 | 0.709 |  |  |  |
| Congruency | 0.021 | 0.008 | 2.75 | 0.698 | 7.58 | 1 | 0.006 |
| Word position | 0.044 | 0.003 | 16.20 | 0.687 | 260.71 | 1 | < .001 |
| **CW, CW+2, CW+4** |  |  |  |  |  |  |  |
| Intercept | 1.739 | 0.092 | 18.84 | 0.575 |  |  |  |
| Congruency | 0.013 | 0.009 | -1.49 | 0.571 | 2.23 | 1 | 0.136 |
| Word position | 0.007 | 0.002 | 3.51 | 0.573 | 12.30 | 1 | < .001 |
|  |  |  |  |  |  |  |  |
| **Random effects** | Intercept |  |  |  |  |  |  |
| **CW+1, CW+3, CW+5** |  |  |  |  |  |  |  |
| Participant | 0.005 |  |  |  |  |  |  |
| Wordlength | 0.004 |  |  |  |  |  |  |
| **CW, CW+2, CW+4** |  |  |  |  |  |  |  |
| Participant | 0.004 |  |  |  |  |  |  |
| Wordlength | 0.007 |  |  |  |  |  |  |

GLMM was fitted with a Gamma probability distribution and an inverse link function. Coefficients and SE are NOT backtransformed. The predicted RT is the backtransformed RT value in seconds: intercept is the overall mean predicted RT, mean predicted RT for congruency is for moving from congruent to incongruent condition (all other factors remaining constant), and mean predicted RT for word position is for moving from the first word position to the last word position (all other factors remaining constant).
